# An Electronic Teen Questionnaire, the eTeenQ, for Risk Behavior Screening During Adolescent Well Visits in an Integrated Health System: Development and Pilot Implementation

**DOI:** 10.2196/47355

**Published:** 2024-01-12

**Authors:** Shannon Neale, Ella Chrenka, Abhilash Muthineni, Rashmi Sharma, Mallory Layne Hall, Juliana Tillema, Elyse O Kharbanda

**Affiliations:** 1 Department of Family Medicine, Park Nicollet Health Services, Bloomington, MN, United States; 2 Department of Family Medicine, University of Minnesota, Minneapolis, MN, United States; 3 Department of Research, HealthPartners Institute, Bloomington, MN, United States; 4 Department of Primary Care, Fairview Health Services, St Paul, MN, United States

**Keywords:** electronic data capture, data capture, privacy, security, adolescent health, risk behavior screening, screening, acceptance, primary care, adolescent, adolescents, electronic health record, risk behavior, risk, risky, behavior, behaviors, behaviour, behaviours, digital health, eHealth, teenage, teens, teen, teenager, teenagers, children, young adults, youth, online health, web data, online data, user experience, interview, interviews, qualitative

## Abstract

**Background:**

Screening for risk behaviors is a routine and essential component of adolescent preventive health visits. Early identification of risks can inform targeted counseling and care. If stored in discrete fields in the electronic health record (EHR), adolescent screening data can also be used to understand risk behaviors across a clinic or health system or to support quality improvement projects.

**Objective:**

Goals of this pilot study were to adapt and implement an existing paper adolescent risk behavior screening tool for use as an electronic data capture tool (the eTeenQ), to evaluate acceptance of the eTeenQ, and to describe the prevalence of the selected risk behaviors reported through the eTeenQ.

**Methods:**

The multidisciplinary project team applied an iterative process to develop the 29-item eTeenQ. Two unique data entry forms were created with attention to (1) user interface and user experience, (2) the need to maintain patient privacy, and (3) the potential to transmit and store data for future use in clinical care and research. Three primary care clinics within a large health system piloted the eTeenQ from August 17, 2020, to August 27, 2021. During preventive health visits for adolescents aged 12 to 18 years, the eTeenQ was completed on tablets and responses were converted to a provider display for teens and providers to review together. Responses to the eTeenQ were stored in a REDCap (Research Electronic Data Capture; Vanderbilt University) database, and for patients who agreed, responses were transferred to an EHR flowsheet. Responses to selected eTeenQ questions are reported for those consenting to research. At the conclusion of the pilot, the study team conducted semistructured interviews with providers and staff regarding their experience using the eTeenQ.

**Results:**

Among 2816 adolescents with well visits, 2098 (74.5%) completed the eTeenQ. Of these, 1811 (86.3%) agreed to store responses in the EHR. Of 1632 adolescents (77.8% of those completing the eTeenQ) who consented for research and remained eligible, 1472 (90.2%) reported having an adult they can really talk to and 1510 (92.5%) reported feeling safe in their community, yet 401 (24.6%) reported someone they lived with had a gun and 172 (10.5%) reported having had a stressful or scary event that still bothered them. In addition, 157 (9.6%) adolescents reported they were or wondered if they were gay, lesbian, bisexual, pansexual, asexual, or other, and 43 (2.6%) reported they were or wondered if they were transgender or gender diverse. Of 11 staff and 7 providers completing interviews, all felt that the eTeenQ improved confidentiality and willingness among adolescents to answer sensitive questions. All 7 providers preferred the eTeenQ over the paper screening tool.

**Conclusions:**

Electronic capture of adolescent risk behaviors is feasible in a busy clinic setting and well accepted among staff and clinicians. Most adolescents agreed for their responses to risk behavior screening to be stored in the EHR.

## Introduction

Adolescence is a period of rapid and complex transitions. Biological maturity often precedes psychosocial maturity and the choices made during this period can have both immediate and long-term health consequences. While many adolescent risk behaviors are transient or experimental, habits and unhealthy coping strategies with origins in adolescence may persist into adulthood [1,2]. In addition, limit-testing behaviors explored during adolescence can increase risk for injury and can contribute to long-term morbidity and mortality [3].

Routine risk behavior screening of adolescents is an important part of providing comprehensive and equitable care to this age group and it is a recommended best practice by the American Academy of Pediatrics [4,5]. Early recognition and response to high-risk adolescent behaviors can help to maintain youth on a healthy trajectory. Standardized questionnaires assessing adolescent risk behaviors have been found to help shift the focus of primary care visits from data gathering to discussion and counseling around sensitive topics. Furthermore, use of standardized questionnaires can improve organization and efficiency during the visit [6].

Many primary care practices rely on paper adolescent screening tools. However, these tools are often not completed by patients and results of the paper screening are inconsistently recorded in the electronic health record (EHR), making it difficult to monitor individual or population-level risk behaviors over time [7]. Electronic data capture for adolescent risk behavior screening has several potential advantages over paper screening and is generally preferred by adolescents [8-10], including those engaging in high-risk behaviors [9,11].

Nevertheless, barriers to adoption of electronic risk behavior screening tools remain, including clinical and institutional inertia and concerns regarding confidentiality of patient-reported risk behaviors collected on electronic tablets or similar devices [12]. In addition, risk behavior screening in primary care through paper or electronic methods may prolong visits and crowd out time for addressing other acute health issues.

In this pilot project, we adapted a paper-based adolescent risk behavior screening tool, the Adolescent and Young Adult Teen Questionnaire [13], which was developed by the Minnesota Department of Health and is currently in use in our health system as a paper screening tool at all primary care clinics, for use as an electronic data capture tool, the eTeenQ. We then pilot-tested the eTeenQ at 3 primary care clinics within our large, integrated, Midwestern health system, with goals of evaluating patient and clinician acceptance of the eTeenQ and describing the prevalence of selected adolescent risk behaviors as reported through the eTeenQ.

## Methods

### Adaptation of the Paper Risk Screening Tool for Electronic Data Capture as the eTeenQ

The multidisciplinary project team, which included 2 primary care physicians, 1 project manager, and 2 members of the HealthPartners Institute software engineering team, applied an iterative process over a 4-month period to adapt a paper risk behavior screening tool, the Adolescent and Young Adult Teen Questionnaire, for use as an electronic data capture form to be completed at the point of care on an electronic tablet. Two unique data entry forms were created with attention to (1) user interface and user experience (UI/UX), (2) the need to maintain patient privacy, and (3) the potential to transmit and store data for future use in clinical care and research. Additional considerations that affected the overall design of the eTeenQ included the need for the system to be stable, process responses quickly, and require limited ongoing maintenance. The overall architecture of the eTeenQ is shown in Multimedia Appendix 1.

The first electronic, web-based form created allowed clinic staff at check-in to enter the patient medical record number (MRN) and then to confirm the patient identity (Multimedia Appendix 2). The GetPatientDemographics application programming interface (API) from Epic Systems was used to identify the correct patient. The second electronic, web-based form created enabled adolescent patients to complete the 29-item fixed-response questions on safety, physical activity, diet and body image, school, self-harm, gender identity, sexual identity, and sexual activity that comprise the Adolescent and Young Adult Teen Questionnaire (Multimedia Appendix 3). The second form contained additional questions for adolescents to consent for their survey responses to be used for research and stored in the EHR, as described below. The eTeenQ forms were developed in REDCap (Research Electronic Data Capture; Vanderbilt University). REDCap is a secure web application used for building and managing online databases and surveys [14].

At the point of care, responses to the eTeenQ are converted to a provider display and adolescents and their primary care providers review responses together on the tablet during the visit. As shown in Multimedia Appendix 4, the provider display highlights eTeenQ “positive screens” or responses requiring attention during the visit. In real time, eTeenQ responses are stored in a REDCap secure external database [14]. For patients who consented to have their data stored in the EHR, after completing the eTeenQ and pressing Submit, a copy of their eTeenQ responses was automatically saved. The SetSmartDataValues API from Epic Systems was used to securely transfer the data from REDCap to custom discrete data elements created in the EHR for the project.

Additional security was integrated to the build of the tool to prevent the use of the tool outside the health system’s intranet. Software use and data transfer were closely monitored throughout the project, and the tablet firmware was maintained.

### Setting and Population for Pilot Implementation

HealthPartners Care Group includes a multispecialty group practice of more than 1800 physicians, 8 hospitals, 55 primary care clinics, 22 urgent care locations, 24 dental clinics, and numerous specialty practices in Minnesota and western Wisconsin. The care group uses a common EHR (Epic; Epic Systems Corporation). Adolescents aged 12 to 18 years receive primary care within the care group from physicians trained in pediatrics, family medicine, or internal medicine, as well as from advanced practice providers, including nurse practitioners and physician assistants.

In June 2018, all 55 primary clinics within HealthPartners Care Group implemented comprehensive screening for well-being and risk behaviors among adolescents aged 12 to 18 years during well visits. At the time of check-in, patients and their parents were each handed a letter that described policies related to confidential care for adolescents and notified them that the adolescent would be completing a paper questionnaire for teens (based on the Adolescent and Young Adult Teen Questionnaire [13] developed by the Minnesota Department of Health). The letter provided at check-in also advised the adolescents and their parents that during the visit the parent would be asked to leave the room so the provider could review responses to the paper questionnaire in private with the adolescent. After the visit, the paper questionnaire was shredded, and it was at the discretion of the clinician to document any patient responses or discussion related to the risk behaviors identified in the EHR.

This pilot implementation of the eTeenQ took place at 3 clinics within the HealthPartners Care Group. Clinic A, located in a small town in Minnesota, had 27 pediatricians participate during the period from January 18, 2021, to August 27, 2021; clinic B, located in a metropolitan area of St Paul, Minnesota, had 5 pediatricians participate during the period from January 18, 2021, to August 27, 2021; clinic C, located in a Minneapolis suburb, had 3 pediatricians and 8 family medicine clinicians participate during the period from August 17, 2020, to August 27, 2021.

All patients aged 12 to 18 years and presenting for a preventive health or well visit with a participating primary care provider at a pilot site during the study period were eligible to participate. Eligible visits were identified through current procedural terminology (CPT) codes for these preventive health visits: 99384, 99394, 99385, and 99395; they were also identified through the *International Classification of Diseases, 10th Revision–Clinical Modification (ICD-10-CM)* codes Z00.121, Z00.129, Z00.00, and Z00.01.

### Training and Support

All staff and providers at the 3 pilot sites attended lunchtime in-person or virtual training(s) regarding the pilot test and implementation of the eTeenQ. At each clinic, an operational staff member and a provider were designated as site champions and points of contact for the research team. Throughout the pilot study, research staff regularly connected by phone, email, or in person with the clinic site champions to obtain informal feedback about the intervention, including identification and triage of any challenges with workflow, technology, or confidentiality.

### Pilot Clinic Workflow for the eTeenQ

Successful implementation of the eTeenQ required careful attention to clinic workflow. Electronic tablets were preloaded with a link to the eTeenQ data capture form. As shown in Multimedia Appendix 2, at the time of registration staff opened this link and entered the patient’s MRN. After a verification process to confirm that the MRN corresponded to the correct adolescent patient, the tablet was handed to the patient along with instructions to complete the eTeenQ on their own, without input from parents or other guardians (Multimedia Appendix 2). After completing the eTeenQ, patients completed 2 additional questions regarding permission to import the eTeenQ data into the EHR and permission for responses to the eTeenQ to be accessed for research. Patients were instructed to hand the electronic tablet to the rooming staff so it could be reviewed by the clinician in advance and discussed during the confidential portion of the visit.

Prior to entering the patient room, the clinician reviewed the data on the tablet. Any survey responses that would generally require attention during the visit were highlighted on the provider display in order to facilitate efficiency (Multimedia Appendix 3).

### Evaluating Use of the eTeenQ and Responses

Use of the eTeenQ was assessed by comparing the total number of adolescent preventive health visits at the 3 participating sites during the pilot period to the number of completed eTeenQ surveys stored in the REDCap database. For those consenting for their data to be used in research, eTeenQ responses were linked to administrative data (eg, age, sex, race/ethnicity, and insurance type) as recorded in the EHR. Selected responses were compared by age group (12-14 years vs 15-18 years) with the chi-square test with a 2-sided *P*<.05 as the threshold for significance. All analyses were conducted in SAS (version 9.4; SAS Institute).

### Obtaining Feedback From Providers and Clinic Staff at Pilot Sites

At the conclusion of the pilot, the study primary investigator (SN) conducted brief in-person or virtual semistructured interviews with participating clinic providers and staff to understand their experiences using the eTeenQ. Questions for clinic staff included the following: “How did this pilot go?” “How did it work to hand out the tablets at the front desk to adolescents to complete the eTeenQ before the visit?” “Can you tell me about any difficulties with the technology or workflow?” “What ideas do you have about improvements we should make to the workflow or technology before spreading across primary care?” and “Do you have any additional feedback regarding this pilot?”

Questions for providers included the following: “How did this pilot go?” “Can you tell me about any difficulties with the technology or workflow?” “How did it work to review the Teen Questionnaire on a tablet?” “As compared to prior to the pilot, how did use of the eTeenQ impact visit efficiency?” “As compared to prior to the pilot, how did use of the eTeenQ impact quality of care?” “As compared to prior to the pilot, how did use of the eTeenQ impact adolescent clinician communication?” “As compared to prior to the pilot, how did use of the eTeenQ impact parent-clinician communication?” “As compared to prior to the pilot, how did use of the eTeenQ impact adolescent confidentiality?” “Did you make changes to clinical care or documentation as a result of the data reviewed on the tablet during the visit?” “Were you able to find the results of the eTeenQ in Epic, after the visit?” “How satisfied are you with electronic capture of eTeenQ data on a tablet?” (3-point scale for responses) and “Do you have a preference for how adolescent risk behavior screening should be administered at your clinic in the future?”

The study primary investigator took notes during the semistructured interviews. These notes were reviewed by the full study team to identify common themes regarding perceptions, preferences, and actual use of the eTeenQ.

### Ethical Considerations

This study was reviewed and approved by the HealthPartners Institutional Review Board (A19-123). Implementation of the eTeenQ at pilot sites was approved with a waiver of informed consent. Adolescent consent for eTeenQ survey responses to be used for research and to be stored in the EHR was obtained as described below.

Adolescents consented for their eTeenQ responses to be used for research by reading the following prompt and then answering the consent question on the tablet:

We are asking all teens who complete the Teen Questionnaire on a tablet for permission to group their answers together in a large database. This data will be used to better understand the health of teens in our clinics and to improve care for teens in the future. We will not include your name or other information about you in the database. This study is voluntary. That means you can tell us that you do not want us to use your answers to the questionnaire for research. This will not affect your care today or in the future. We expect up to 1000 adolescents to participate in this study.

Do you give permission to use your answers to this questionnaire for research?

Adolescents consented for their eTeenQ responses to be stored in their EHR by reading the following prompt and then answering the consent question on the tablet*:*

The information in this questionnaire is confidential. It will be used by the doctors and nurses taking care of you to provide the best care possible. In the occasion that your parent or guardian requests a copy of your entire medical record, it is possible they may see the answers you provided on this form. If you report that you intend to harm yourself or to harm others, we are required to intervene on your behalf.

Do you give permission to save this information in your medical record so that it can be used to help take care of you at future medical appointments?

## Results

### Evaluating Use of the eTeenQ

**Figure 1.**
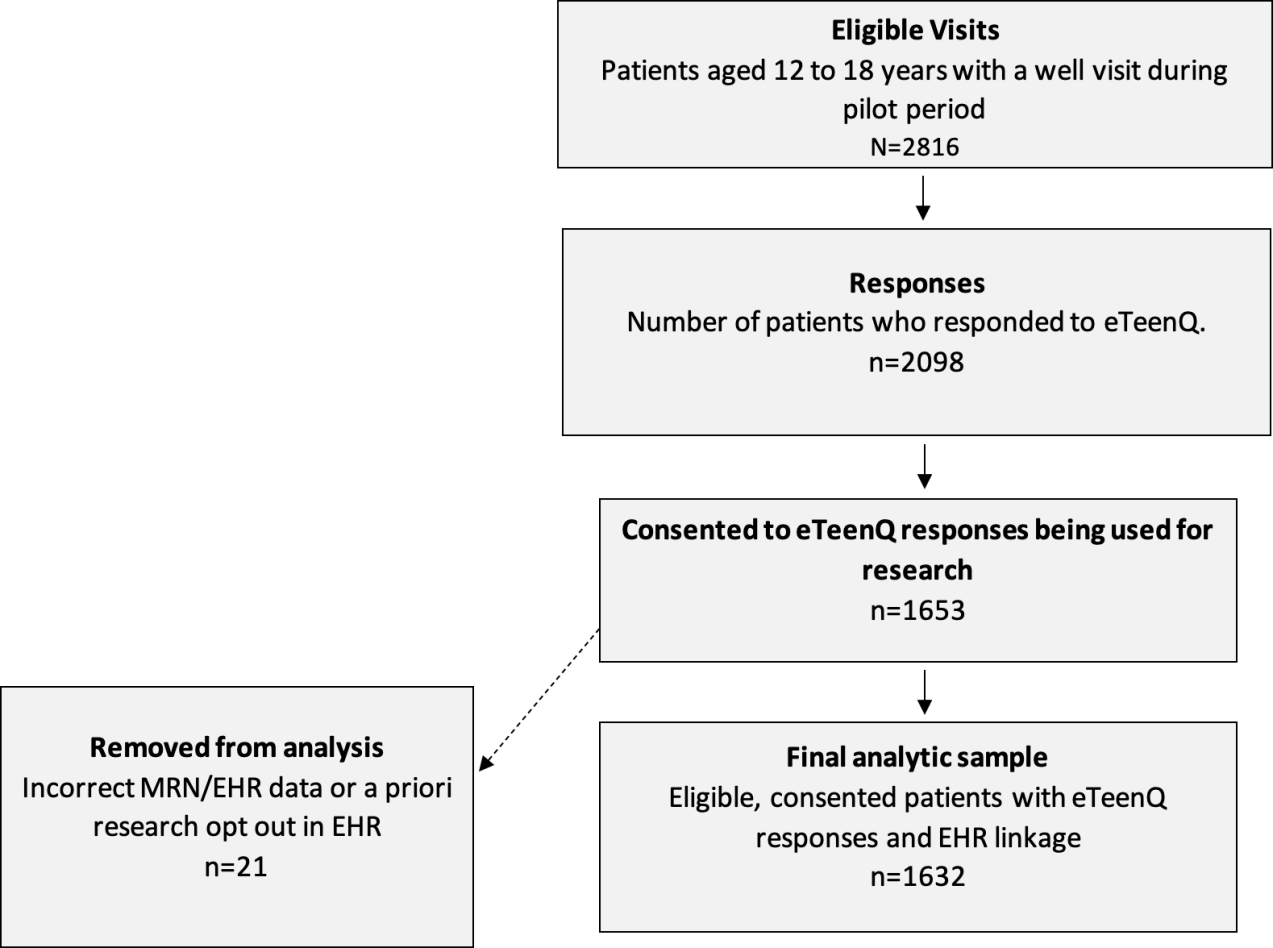
Flowsheet of study eligibility and consent for data to be used in research. EHR: electronic health record; MRN: medical record number.

During the pilot period, among 2816 eligible adolescents with well visits, 2098 (74.5%) completed the eTeenQ. Of the 2098 adolescents who completed the eTeenQ, 1653 (78.8%) consented to have their data used for research and 1811 (86.3%) agreed to have their data stored in the EHR. After excluding 21 responses due to incorrect MRN linkage to the EHR or because the patient had an a priori research opt-out recorded in their EHR, the final analytic sample included 1632 adolescents; 818 (50.1%) were female and the mean age was 14.5 (SD 1.8) years. (Figure 1, Table 1)

**Table 1.** Characteristics of sample (n=1632).

Characteristics		Values
**Site, n (%)**		
	Clinic A	997 (61.1)
	Clinic B	231 (14.2)
	Clinic C	404 (24.8)
Age (years), mean (SD)		14.5 (1.8)
**Sex, n (%)**		
	Female	818 (50.1)
	Male	814 (49.9)
**Race/ethnicity, n (%)**		
	White	1224 (75)
	Black/African American	139 (8.5)
	Asian	72 (4.4)
	Native Hawaiian or Pacific Islander	60 (3.7)
	American Indian or Alaska Native	4 (0.3)
	Other (or multiple)	74 (4.5)
	Unknown	59 (3.6)
	Hispanic	70 (4.3)
**Insurance type, n (%)**		
	Commercial	1284 (78.7)
	Public	340 (20.8)
	Missing	8 (0.5)

Overall data quality was good. Of 1632 respondents in the analytic sample, 1562 (95.7%) responded to all 29 questions in the eTeenQ. The most common question skipped was “How often do you use marijuana?” but this was only left incomplete for 10 (0.6%) respondents.

Across all 1632 teen respondents, 1472 (90.2%) reported having an adult they can really talk to and 1510 (92.5%) reported feeling safe in their community, yet 401 (24.6%) reported someone they lived with had a gun and 172 (10.5%) reported having had a stressful or scary event that still bothered them. In addition, 263 (16.1%) reported missing 7 or more days of school and 196 (12%) reported their grades were worse than they used to be. In addition, 157 (9.6%) responded they were or wondered if they were gay, lesbian, bisexual, pansexual, asexual, or other, and 43 (2.6%) reported they were or wondered if they were transgender or gender diverse. Risk behaviors were more common among older adolescents (aged 15-18 years; n=774) as compared to younger adolescents (aged 12-14 years; n=858). For example, 137 (17.7%) of older adolescents reported ever having had any kind of sex (with anyone of any gender) as compared to 9 (1%) of younger adolescents (*P*<.001). Similarly, 133 (17.2%) of older adolescents reported ever having used alcohol and 94 (12.1%) reported ever having used marijuana, as compared to 25 (2.9%) and 11 (1.3%), respectively, reporting use among younger adolescents (*P*<.001 for comparisons by age group).

### Feedback From Providers and Clinic Staff at Pilot Sites

Across the 3 pilot clinics, 18 providers and clinic staff provided feedback through semistructured interviews. All felt that their adolescent patients liked using the tablets for completing the eTeenQ. They believed that patients were more honest with their responses using the tablet and noted more “positive screens” than when screening for adolescent risk behaviors on paper. They felt the tablets enhanced privacy and they particularly liked the provider display on the tablet that highlighted the “positive screens” or topics to address during the visit.

Challenges with the eTeenQ reported during interviews included isolated interruptions of Wi-Fi connectivity and confusion about which tablet belonged to which adolescent when 2 or more siblings with well visits were in the same examination room. In addition, adolescents occasionally clicked past the provider display screen, and the provider was then unable to review the eTeenQ responses on the tablet. Despite these occasional minor difficulties, primary care providers felt that the eTeenQ improved the quality of care they were able to provide and enhanced adolescent confidentiality. When asked their screening preference going forward, paper vs electronic, all respondents chose electronic.

## Discussion

### Principal Results

In this pilot study conducted in 3 busy community-based clinics within a large health system, we demonstrated that a paper adolescent risk screening tool can be converted for use as an electronic form; that adolescents were generally adherent to completing electronic risk screening on tablets at the time of preventive health visits, with a majority agreeing to have their responses stored in the EHR; and that implementation of the eTeenQ was feasible and well accepted by providers. Furthermore, the conversion of adolescent questionnaire responses to an intuitive provider display may have improved identification of risk behaviors requiring attention or further discussion during the visit. Storage of eTeenQ responses in a discrete field in the EHR can allow clinicians or researchers to evaluate adolescent risk behaviors across a clinic or geographic region, and ultimately can be used to design and implement targeted quality improvement projects.

### Limitations

Several limitations to this pilot study should be noted. First, the participating clinics were not randomly selected. These clinics were motivated sites and had site champions who were engaged partners throughout the pilot. The successful adaptation and implementation of the eTeenQ at our pilot sites may not be generalizable to other clinics or health systems. A second limitation was that while adolescents complete several questionnaires at their preventive health visits, due to limitations in scope and budget, in our pilot only the eTeenQ was completed electronically. Thus, teens were filling out forms both electronically and on paper, which was cumbersome for staff and patients. Third, as a pilot project, we were not able to optimize all aspects of UI/UX, and the transfer of the data from REDCap to the EHR required a manual trigger following patient consent. If widely implemented, we would encourage additional modifications to the display and updates to the architecture to allow eTeenQ responses to flow seamlessly, in real time, into the EHR, and the incorporation of all adolescent screening tools for completion through electronic data capture. Further enhancements should also support completion of risk behavior screening in the days prior to preventive health visits. Fourth, as a small pilot study, our assessment of provider and staff acceptance of the eTeenQ was based on brief semistructured interviews and did not include formal qualitative analyses.

### Comparisons With Prior Work

Findings from this pilot study were consistent with prior research, which has demonstrated that adolescents appear to more accurately report and be more willing to disclose sensitive information when questioned electronically vs on paper or in person [15,16]. In a pilot study conducted in 2015 in an academic adolescent clinic in Seattle, teens aged 13 to 18 years reported they preferred an electronic screen to a paper version. Prior studies have also noted that adolescents also perceive their visits as more confidential, feel they are listened to more carefully, and report they are more satisfied with their visit when computerized screening is used, as compared to other approaches to adolescent risk behavior screening [17]. Our study adds to the literature, as we conducted this work outside of an academic setting in 3 community-based clinics.

A potential benefit of electronic data capture is that forms can be easily modified and can include additional skip patterns or branching logic to support additional targeted data collection. For example, for those responding “yes” or “sometimes” to the eTeenQ single screening question regarding gender identity, “Are you or do you wonder if you are transgender or gender diverse?” in future iterations additional questions could then display allowing the patient to specify their gender identity [18].

We are not aware of prior research on adolescent preferences for storing sensitive information in the EHR. Prior to conducting this pilot, health system leaders had assumed that adolescent patients would not want their responses to adolescent risk screening stored in the EHR, as there would be a potential risk for disclosure to parents or others accessing their medical records. As such, we were surprised to find that 86% of adolescents consented to store their eTeenQ responses in the EHR. Recording of eTeenQ responses in discrete fields in the EHR is critical for follow-up of risk behaviors at future visits. If not documented, important health information revealed through risk behavior screening may be lost and not available at a patient’s next medical encounter. In addition, adolescents may assume that communication between care teams occurs in the EHR and may not reveal vital sensitive information during a subsequent visit.

### Conclusions

The use of electronic data capture for adolescent risk screening in primary care is feasible for collecting sensitive information in busy, community-based primary care settings. Most adolescents were agreeable to having their data stored in the EHR, and staff and primary care providers preferred electronic to paper screening. Providers felt that electronic screening enhanced confidentiality and that the eTeenQ improved the quality of care overall.

## Supplementary material

10.2196/47355 Multimedia Appendix 1 Architecture of the eTeenQ and data transfer.

10.2196/47355 Multimedia Appendix 2 Check-in workflow for the eTeenQ.

10.2196/47355 Multimedia Appendix 3 First 7 questions of the eTeenQ for adolescents to complete, as displayed on a tablet.

10.2196/47355 Multimedia Appendix 4 Provider display of eTeenQ responses, with positive results highlighted in blue.

## Abbreviations

API: application programming interface
CPT: current procedural terminology
EHR: electronic health record
*ICD-10-CM*: *International Classification of Diseases, 10th Revision–Clinical Modification*
MRN: medical record number
REDCap: research electronic data capture
UI/UX: user interface and user experience
