# Supplementary figures and images for: An Electronic Teen Questionnaire, the eTeenQ, for Risk Behavior Screening During Adolescent Well Visits in an Integrated Health System: Development and Pilot Implementation
Source: JMIR Pediatr Parent. 2024 Jan 12;7:e47355. doi: 10.2196/47355 (PMC10973640; doi:10.2196/47355)

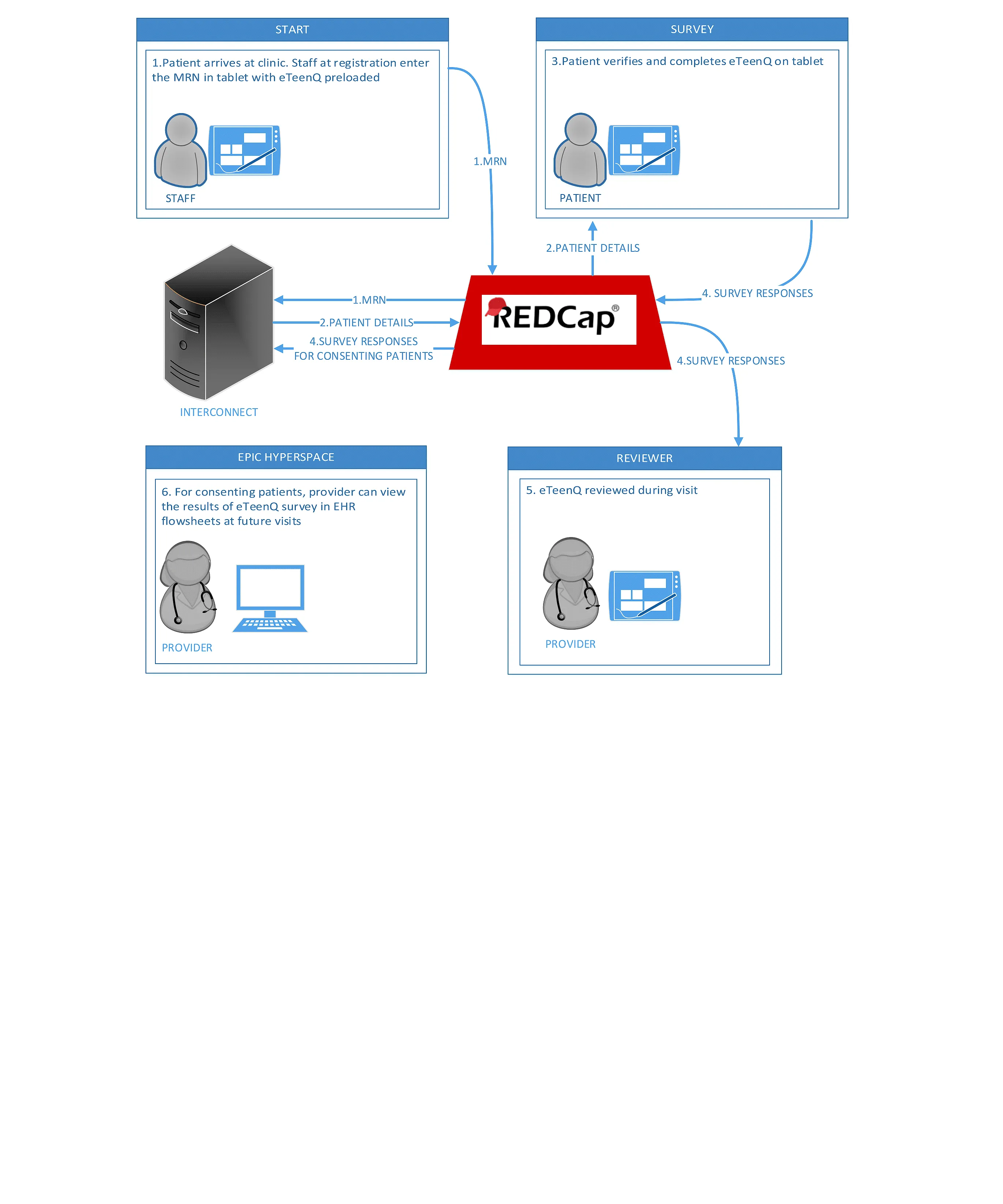

Supplement: Multimedia Appendix 1 [file pediatrics-v7-e47355-s001.png]

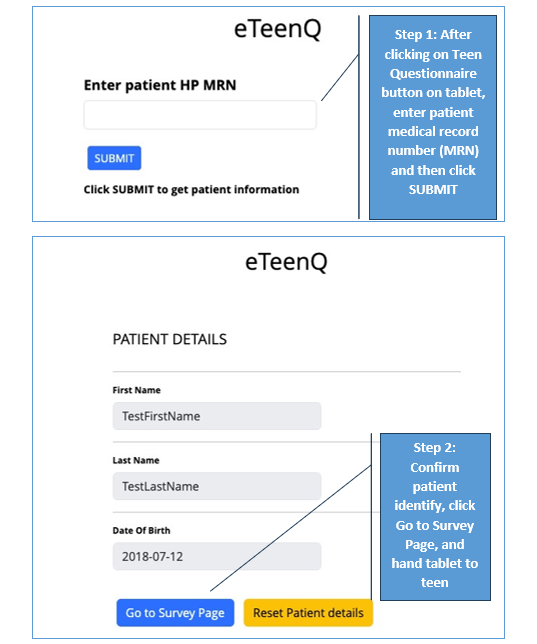

Supplement: Multimedia Appendix 2 [file pediatrics-v7-e47355-s002.png]

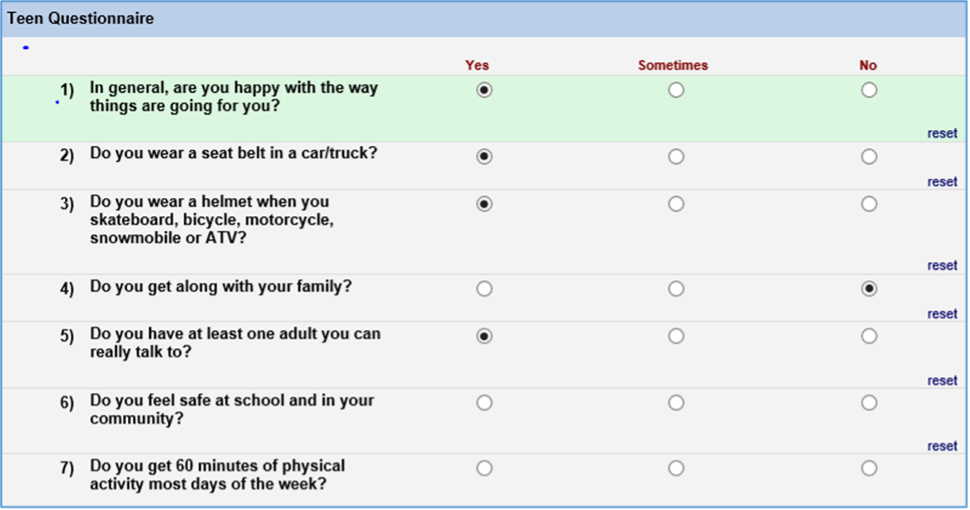

Supplement: Multimedia Appendix 3 [file pediatrics-v7-e47355-s003.png]

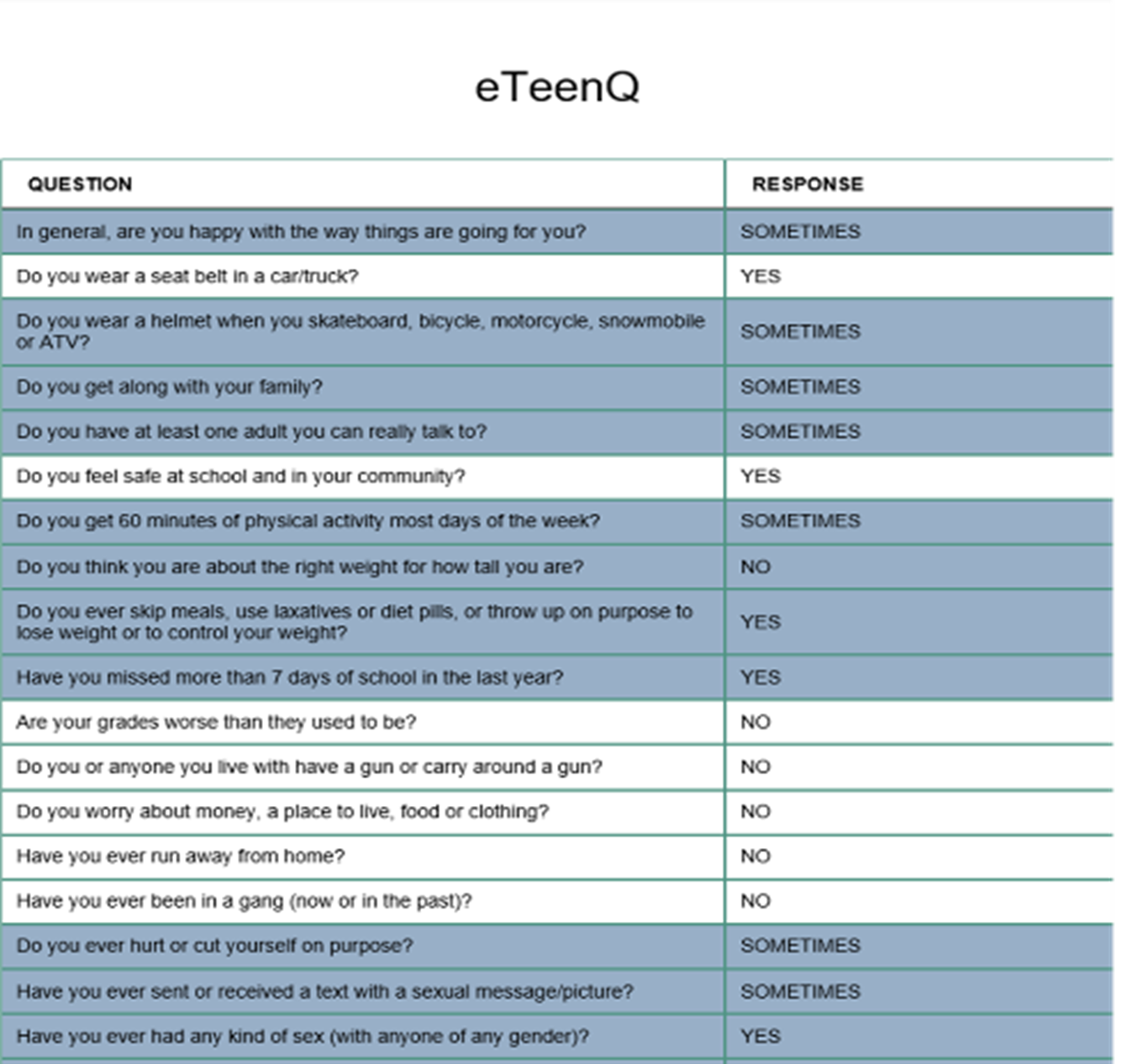

Supplement: Multimedia Appendix 4 [file pediatrics-v7-e47355-s004.png]
